# Animal Toxins and Their Advantages in Biotechnology and Pharmacology

**DOI:** 10.1155/2014/951561

**Published:** 2014-05-22

**Authors:** S. L. Da Silva, E. G. Rowan, F. Albericio, R. G. Stábeli, L. A. Calderon, A. M. Soares

**Affiliations:** ^1^Department of Chemistry, Biochemistry and Bioprocess Engineering, Federal University of São João del-Rei, Campus Alto Paraopeba, 34420-000 Ouro Branco, MG, Brazil; ^2^Strathclyde Institute of Pharmacy and Biomedical Sciences, University of Strathclyde, Glasgow G4 0NR, UK; ^3^Institute for Research in Biomedicine, Barcelona Science Park, University of Barcelona, 08028 Barcelona, Spain; ^4^CIBER-BBN, Barcelona Science Park, University of Barcelona, 08028 Barcelona, Spain; ^5^Department of Organic Chemistry, University of Barcelona, 08028 Barcelona, Spain; ^6^Center of Biomolecules Study Applied to Health, Fiocruz Rondônia, Oswaldo Cruz Foundation, Medicine Department, Federal University of Rondônia, 76812-245 Porto Velho, RO, Brazil

Biodiversity provides a huge source of new chemical entities that could be useful for the development of new therapies. An example is venomous animals, which produce venoms that share common features, such as compositions characterized by a complex combination of bioactive proteins and peptides with wide structural diversity. The biological activities of these compounds are selective and specific and are currently dependent on the synergic action of several components. Thus, animal venoms are important tools for carrying out biochemical, physiological, and pathological studies, as well as for the development of new biotechnological and pharmaceutical products.

The modern approach used to characterize various compounds from animal venoms, using advanced proteomic and genomic tools, has been denominated “venomics.” Modern technologies currently available in several research laboratories have allowed scientists to obtain the identification and functional-structural characterization of hundreds of toxins from snakes, scorpions, spiders, anurans, and marine invertebrates, presenting a high diversity of pharmacological activities. Venomics delineates a scientific area of high complexity that is increased by the diversity of venom proteins and the influence imposed by age, geographic location, ontogenetic variation, feeding, and individual intrinsic characteristics that produce intraspecific variations.

The study of toxins and their isoforms has allowed for a better understanding of the toxic mechanisms of envenomation. The identification/characterization of different isoforms of venom toxins, along with the search for natural and synthetic inhibitors, such as monoclonal or polyclonal antibodies, and molecules with different chemical properties, such as heparin, clotting factors, and plant extracts, has increased the possibility of using these agents as more effective therapeutic alternatives.

More recently, venomics has focused on better understanding of the clinical aspects of human envenomation, the mechanism of action of venoms and their toxins, the prospection of toxins with biotechnological/pharmaceutical potential, and the development of novel antivenom serum and alternative therapies for envenomation.

Among venom toxic components, phospholipases A_2_, L-amino acid oxidases, and proteolytic enzymes, which are classified as metalloproteases and serine proteases, are able to disrupt the human haemostatic system through different mechanisms. Nonenzymatic proteins, such as disintegrins and bradykinin-potentiating peptides (BPPs), and other components, such as carbohydrates, lipids, ions, biogenic amines, nucleotides, and free amino acids, are also components of venom.

This special issue presents three revisions and eight original papers addressing different aspects of animal venom components, their inhibitors, and possible biotechnological applications. This is relevant considering that snake envenomation is still an important public health issue in many countries, especially tropical ones.

M. B. A. Carvalho et al. present, herein, a mini-review on phospholipase A_2_ inhibitors from different sources, such as Brazilian plants and their chemical synthesis. These authors report on the antivenom effect of different plant extracts used by traditional communities. These extracts have been scientifically validated through the phytochemical identification and characterization of some isolated inhibitors from these species, leading to the development of new therapeutic alternatives for envenomation. L. F. M. Izidoro et al. reviews L-amino acid oxidases isolated from snake venoms, focusing on their biochemical, biological, and enzymatic characteristics and discussing the importance of hydrogen peroxide production, antigenicity, molecular and structural characteristics, and biotechnological and pharmacological applications of LAAOs focusing on their antibiotic potential. Finally, in the third revision paper of this special issue, L. A. Calderon et al. discussed the continuous search for new bioactives in order to develop new anticancer drugs. In this review, the authors describe the potential of proteins and peptides from snake venoms, which show* in vitro* and* in vivo* cytotoxicity against cancer cells, for the development of more efficient and safe therapies.

Among the eight original papers, three of them describe the isolation and biochemical characterization of phospholipases A_2_ and basic and acidic phospholipase A_2_ homologs from the venoms of* Bothrops atrox* (J. L. Furtado et al., 2014),* Bothrops mattogrossensis* (A. A. de Moura et al., 2014), and* Crotalus oreganus abyssus* (W. Martins et al., 2014), which show inflammatory, leishmanicidal, and antitumoral activities, respectively.

With regard to the search for complementary alternatives for conventional serum therapies, E. C. De Oliveira et al. describe the antivenom potential of* Manilkara subsericea* extract against* Lachesis muta* venom, and C. L. S. Guimarães et al. relate the biotechnological application of polyclonal antibodies produced using alkylated myotoxic phospholipases A_2_ from* Bothrops jararacussu* venom with reduced myotoxic activity.

Finally, three papers report on proteases from animal venoms, such as snakes and spiders and their biotechnological potential. F. D. Torres-Huaco et al. and K. D. Zaqueo et al. describe the biochemical characterization of two thrombin-like enzymes (SVTLE) isolated from* Lachesis muta* and* Bothrops pirajai *venoms, respectively. Both enzymes show blood-clotting activity, indicating a thrombolytic potential. In contrast, G. Gimenez et al. demonstrate the proteolytic activity of venom from the social spider* Parawixia bistriata* and its possible biotechnological application as an insecticide against* Aedes aegypti*.

Research regarding animal venoms generally focuses on the clinical features of human envenomation, the relationship between structure and function of animal toxins, bioprospection of toxins with potential for the development of therapies, diagnostic kits for human diseases, and alternative or complementary therapies for envenomation. Therefore, research involving the biologically active components of animal venoms could result in the direct or indirect use of components of venoms during the development of synthetic models in order to obtain new drugs.

Thus, understanding the mechanism of action responsible for venoms' toxic and pharmacologic effects, along with their biochemical and structural characterizations, is essential in order for them to be used as biotechnological prototypes in a range of medical and scientific areas.

*S. L. Da Silva* *E. G. Rowan* *F. Albericio* *R. G. Stábeli* *L. A. Calderon* *A. M. Soares*

